# Eosinopenia predicts poor outcomes in patients with lung cancer with the omicron variant of COVID-19

**DOI:** 10.3389/fmed.2025.1583843

**Published:** 2025-07-09

**Authors:** Xiao Hu, Jie Tan, Rumei Luan, Dongyan Ding, Ming Yue, Junling Yang, Qianfei Xue

**Affiliations:** ^1^Department of Respiratory Medicine, The Second Hospital of Jilin University, Changchun, China; ^2^Shandong First Medical University Affiliated Provincial Hospital, Jinan, Shandong, China; ^3^Department of Pulmonary and Critical Care Medicine of Jiangbei Campus, The First Affiliated Hospital of Army Medical University, Chongqing, China; ^4^Department of Internal Medicine, Hospital of Jilin University, Changchun, China

**Keywords:** omicron, COVID-19, lung cancer, eosinopenia, outcomes

## Abstract

**Background:**

Lung cancer is among the malignancies most vulnerable to coronavirus disease 2019 (COVID-19). Eosinophils have anti-tumor and antiviral effects. Since November 2021, the omicron variant of COVID-19 has become a topic of concern; however, the impact of eosinophils on the severity and outcomes of patients with lung cancer with omicron remains uncertain. This study aimed to utilize eosinophils to predict patient outcomes and guide the prevention and monitoring of omicron.

**Methods:**

This study performed an analysis of 284 patients with lung cancer who were hospitalized in the second hospital of Jilin University, of whom 83 patients were confirmed to have omicron infection. Depending on the eosinophil counts, patients were divided into two groups: low and high eosinophil counts. The relationship between eosinophil counts and severity and outcomes was then analyzed.

**Results:**

We found that omicron, especially severe-to-critical omicron, decreased survival in patients with lung cancer. Patients with omicron had a lower eosinophil count. Patients with eosinopenia (< 0.015 × 10^9^/L) were more likely to have an eastern cooperative oncology group performance status ≥ 2; be undergoing anti-cancer treatment; have comorbidities; and exhibit lower disease control rates, reduced 30-day survival, and shorter overall survival (median 75 days vs. not reached). Multivariate regression analysis revealed that the eosinophil count was an independent predictor of disease severity and survival in patients with lung cancer with omicron.

**Conclusion:**

Eosinopenia correlates with poor outcomes in patients with lung cancer with omicron, and the eosinophil count is an independent indicator for predicting the severity and outcomes in these patients.

## Introduction

The coronavirus disease 2019 (COVID-19), caused by the severe acute respiratory syndrome coronavirus 2 (SARS-CoV-2), has had a remarkable influence on the healthcare system (1). With the emergence of the second and third waves of SARS-CoV-2 (2), multiple variants, including omicron, have emerged, making the management of COVID-19 even more complicated (3).

Cancer and anti-cancer therapy can cause immunosuppression, which may make patients with cancer, compared with those without cancer, more prone to developing infections (4, 5). COVID-19 can damage multiple systems, including the vasculature and respiratory system, and leads to a series of complications, such as pneumonia and thromboembolism (6, 7). Furthermore, among patients with COVID-19, those with cancer are more prone to suffer from infection-related adverse events, such as intensive care unit (ICU) admission, mechanical ventilation, and death, than those without (8, 9). According to the 2020 Global Cancer Assessment report, lung cancer is the leading cause of cancer deaths and the most frequent new cancer in men in China (10). Lung cancer is the second most common cancer type leading to death, second only to patients with hematological malignancies (11).

The risk factors influencing prognosis have received increasing attention. Alongside demographic and oncological characteristics such as age, pneumonia, cancer stage, and eastern cooperative oncology group (ECOG) performance status, immune-related indicators also play a significant role in the survival and prognosis of patients with lung cancer with COVID-19. These indicators include neutrophil counts, procalcitonin levels, C-reactive protein concentrations, and the neutrophil-to-lymphocyte ratio (NLR) (1, 12–14).

Eosinophils are innate immune cells primarily derived from granulocytes in hematopoietic progenitor cells of the bone marrow. Previous eosinophil studies mainly focused on parasitic infections and allergic diseases such as asthma (15, 16). The impact of eosinophils on cancer has also attracted the attention of clinicians. Tumor-associated tissue eosinophilia (TATE) and peripheral blood eosinophilia have been shown to improve the prognosis of various solid tumors, including colon, breast, and lung cancer (17–19). However, Lee et al. (20) indicated that TATE-enriched head and neck squamous cell carcinoma correlates with aggressive pathological features and poor prognosis, underscoring the functional plasticity and heterogeneity of TATE across different tumor types. Additionally, the role of eosinophils in antiviral defense cannot be ignored, as seen in the case of the human influenza virus (21), respiratory syncytial virus (22), and other respiratory viruses. Our previous research found that eosinopenia was associated with increased mortality in patients with COVID-19; however, that study included only non-cancer patients (23). From December 2022 to early 2023, China experienced an epidemic of the COVID-19 omicron variant (24). Thus, this study aimed to investigate the impact of eosinopenia on disease severity and clinical outcomes in lung cancer patients infected with the omicron variant.

## Materials and methods

### Study approval

The studies involving human participants were reviewed and approved by the Ethics Committee of the Second Hospital of Jilin University (Number: 2023-217). Written informed consent for participation was not required from the participants or their legal guardians/next of kin in accordance with national legislation and institutional requirements. The project adhered to Helsinki principles outlined in its declaration.

### Study procedures and participants

This observational study included patients hospitalized in the Second Hospital of Jilin University from December 1, 2022, to February 28, 2023. The inclusion criterion was the pathological diagnosis was lung cancer and age ≥ 18 years old. All patients were tested for COVID-19 (RT-PCR or antigen test of nasopharyngeal swabs) within 0–7 days prior to enrollment (25). Exclusion criteria included lung cancer patients with primary liver or kidney diseases (e.g., cirrhosis, hepatitis, chronic kidney disease), excluded to avoid confounding by preexisting eosinophil dysregulation or treatment-related organ toxicity. Other exclusion criteria were: patients with concurrent other malignant tumors, a history of COVID-19 infection that had resolved by enrollment, missing data on demographics, clinical symptoms, complications, comorbidities, oncological parameters, or laboratory values, and those lost to follow-up.

All variables considered were collected at enrollment or COVID-19 diagnosis and mainly included: (1) demographic characteristics: age, sex, and smoking status; (2) oncological characteristics: ECOG performance status, histological type of lung cancer, cancer stage at enrollment, anti-cancer therapy received within 3 months before enrollment, the number of lines and type of anti-cancer therapy; (3) fever (> 37.5 °C) and respiratory symptoms: cough, expectoration, dyspnea, asthma and chest pain; (4) comorbidities: hypertension, diabetes mellitus, ischemic heart disease and chronic obstructive pulmonary disease (COPD); (5) complications: pneumonia/pneumonitis and venous thromboembolism; (6) laboratory parameters: white blood cell count (WBC), absolute lymphocyte count (ALC), absolute neutrophil count (ANC), eosinophil count, platelet to lymphocyte ratio (PLR), NLR, serum albumin (ALB), lactate dehydrogenase (LDH), hypersensitive C-reactive protein (hs-CRP), β2-microglobulin (β2-MG), and D-dimer; and (7) chest computed tomography scans or chest radiographic images at enrollment.

To initially explore the long-term prognosis of patients with lung cancer with COVID-19, the follow-up period was set at ≥ 6 months after enrollment (26), with a follow-up deadline of September 1, 2023. The primary endpoint was overall survival during the follow-up period, and the secondary endpoints were disease control rate (DCR) and survival rate within 30 days. COVID-19 was diagnosed and classified according to the diagnostic and classification criteria of the Diagnosis and Treatment Plan for Novel Coronavirus Pneumonia (10th edition) (25), and all the enrolled patients with omicron infection were divided into mild cases (with mild clinical symptoms and no signs of pneumonia on imaging); moderate cases (with fever and respiratory symptoms and evidence of pneumonia on imaging); severe cases (patients meeting any of the following criteria: (1) respiratory distress (≥ 30 beats/minute); (2) oxygen saturation at rest ≤ 93%; (3) arterial partial pressure of oxygen (PaO2)/inhaled partial pressure of oxygen (FiO2) ≤ 300 mmHg; (4) cases with progressive aggravation of clinical symptoms, where lung imaging shows a significant 50% progression of the lesion within 24–48 h); and critical cases (patients meeting any of the following criteria: (1) respiratory failure requiring mechanical ventilation; (2) shock; (3) combined with other organ failure requiring ICU monitoring and treatment). DCR was defined as the percentage of patients who achieved complete response, partial response, or stable disease out of the number of evaluable cases after treatment. Progressive disease was defined as the sum of the maximum diameters of the tumor target lesions increasing by at least 20%, or new lesions appearing (27). Patients with lung cancer were followed up according to the European Society for Medical Oncology guidelines (28–30).

### Statistical analysis

SPSS 26.0 and GraphPad Prism 8.0.2 software were used for statistical analyses. The Kolmogorov-Smirnov method was used to analyze the distribution of continuous variables. If the distribution was normal, mean ± standard deviation was used to describe the continuous variables. If the distribution was not normal, the median and interquartile range (IQR) were used for the description, and the Mann-Whitney U test was used for comparison between the two groups. Categorical variables were described using the number of cases and percentages, and the Chi-square tests or Fisher’s exact tests were used for the comparison between groups. The binary logistic regression model was used for the univariate and multivariate analyses (forward likelihood ratio model) of severity. Kaplan-Meier analysis and log-rank test were used to compare the cumulative survival rates. The Cox regression model was used to calculate the hazard ratio (HR) of survival and its associated 95% confidence interval (CI) and perform the univariate and multivariate survival analyses (forward likelihood ratio model). All statistical analyses were based on two-sided hypothesis tests; with α = 0.05 as the test level, *p* < 0.05 was considered statistically significant.

## Results

### Baseline characteristics

**FIGURE 1:**
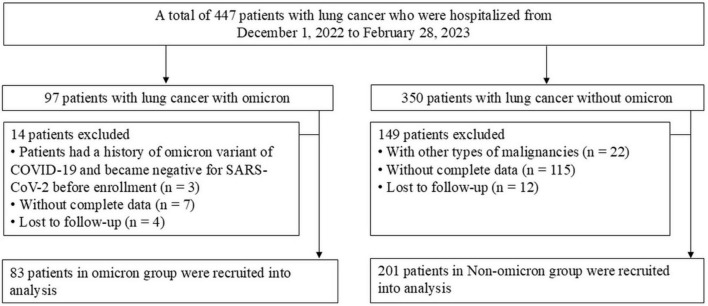
Flow chart for the enrolled patients. COVID-19, coronavirus disease 2019; SARS-CoV-2, severe acute respiratory syndrome coronavirus 2.

A total of 447 patients with lung cancer who were hospitalized were enrolled, of which 163 patients were excluded based on the exclusion criteria (Figure 1).

**FIGURE 2:**
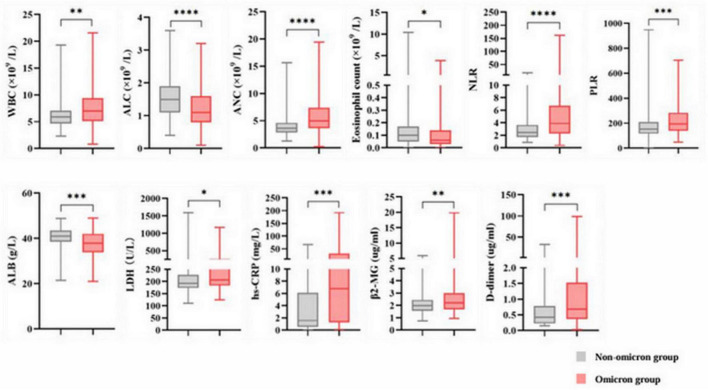
Abnormal baseline laboratory parameters in the non-omicron and omicron groups. * *p* < 0.05; ** *p* < 0.01; *** *p* < 0.001; **** *p* < 0.0001; ns, not significant. WBC, white blood cell count; ALC, absolute lymphocyte count; ANC, absolute neutrophil count; PLR, platelet to lymphocyte ratio; NLR, neutrophil to lymphocyte ratio; ALB, serum albumin; LDH, lactate dehydrogenase; hs-CRP, hypersensitive C-reactive protein; β2-MG, β2-microglobulin.

Our study enrolled 284 patients, including 83 with and 201 without omicron. The median age was 63 years in both groups. Significant differences in ECOG performance status and histological types were observed in the two groups (Table 1). Patients with omicron were more likely to develop pneumonia, fever, and respiratory symptoms, such as cough, expectoration, wheezing, and chest pain, than those without omicron (Supplementary Table S1). Compared with patients without omicron, those with omicron had higher levels of WBC, ANC, PLR, NLR, LDH, hs-CRP, β2-MG, and D-dimer and lower levels of ALC, eosinophil count, and ALB (Figure 2).

**TABLE 1:** Baseline demographics and oncological characteristics of enrolled patients with lung cancer.

Characteristics	Omicron group (*n* = 83)	Non-omicron group (*n* = 201)	All patients (*n* = 284)	*p*-value
Age [years, median (quartile)]	63.0 (57.0∼70.0)	63.0 (56.0∼68.0)	63.0 (57.0∼68.0)	0.195
Sex, n (%)				0.572
Female	37 (44.6%)	97 (48.3%)	134 (47.2%)	
Male	46 (55.4%)	104 (51.7%)	150 (52.8%)	
Smoking status, n (%)				0.506
Former/current	37 (44.6%)	81 (40.3%)	118 (41.5%)	
Never	46 (55.4%)	120 (59.7%)	166 (58.5%)	
ECOG performance status, n (%)				< 0.001
0∼1	57 (68.7%)	179 (89.1%)	236 (83.1%)	
≥ 2	26 (31.3%)	22 (10.9%)	48 (16.9%)	
Histological types, n (%)				0.028
NSCLC	76 (91.6%)	163 (81.1%)	239 (84.2%)	
SCLC	7 (8.4%)	38 (18.9%)	45 (15.8%)	
Cancer stage at enrollment, n (%)				0.501
0- II	28 (33.7%)	76 (37.8%)	104 (36.6%)	
III-IV	51 (61.4%)	120 (59.7%)	171 (60.2%)	
Unknown	4 (4.8%)	5 (2.5%)	9 (3.2%)	
Currently undergoing anti-cancer treatment				0.978
Yes	30 (36.1%)	73 (36.3%)	103 (36.3%)	
No	53 (63.9%)	128 (63.7%)	181 (63.7%)	
Current lines of anti-cancer treatment				0.407
No treatment	51 (61.4%)	124 (61.7%)	175 (61.6%)	
Operative treatment	2 (2.4%)	4 (2.0%)	6 (2.1%)	
Adjuvant/neoadjuvant therapy	6 (7.2%)	5 (2.5%)	11 (3.9%)	
First-line treatment	18 (21.7%)	53 (26.4%)	71 (25.0%)	
Second-line treatment or above	6 (7.2%)	15 (7.5%)	21 (7.4%)	
Current types of anti-cancer treatment				0.330
No treatment	53 (63.9%)	128 (63.7%)	181 (63.7%)	
Operative treatment	2 (2.4%)	7 (3.5%)	9 (3.2%)	
Chemotherapy alone	7 (8.4%)	17 (8.5%)	24 (8.5%)	
Radiotherapy alone	0 (0.0%)	3 (1.5%)	3 (1.1%)	
TKI alone	12 (14.5%)	16 (8.0%)	28 (9.9%)	
Chemotherapy and immune checkpoint inhibitors	6 (7.2%)	27 (13.4%)	33 (11.6%)	
Immune checkpoint inhibitors alone	3 (3.6%)	3 (1.5%)	6 (2.1%)	

ECOG, Eastern Cooperative Oncology Group; NSCLC, non-small cell lung cancer; SCLC, small cell lung cancer; TKI, tyrosine kinase inhibitor.

### ROC curve analysis for eosinophil count to predict severity and mortality

**FIGURE 3:**
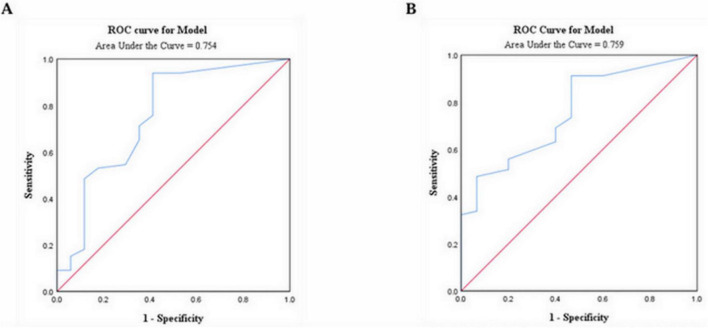
ROC analysis revealed a cut-off value of 0.015 × 10^9^/L for eosinophil count to predict disease severity **(A)** and mortality **(B)** in patients with lung cancer infected with the omicron variant; ROC, receiver operating characteristic.

According to the above results, eosinophils were significantly different in patients with and without omicron. Furthermore, the patients with lung cancer with omicron were divided into a mild-to-moderate group and a severe-to-critical group. Receiver operator characteristic (ROC) analysis found that the cut-off value of eosinophil counts for predicting disease severity and death in patients with lung cancer with omicron was 0.015 × 10^9^/L, and the area under the ROC curve (AUC) was 0.754 (95% CI: 0.608–0.899, *p* = 0.001; Figure 3A) and 0.759 (95% CI: 0.634–0.884, *p* = 0.002; Figure 3B), respectively. Depending on whether the eosinophil count was < 0.015 × 10^9^/L, patients with lung cancer with omicron were divided into low and high eosinophil count groups.

### Associations of eosinophils with demographics, oncology characteristics, and laboratory parameters

We further explored the effects of eosinophils on demographics, oncologic characteristics, and laboratory measures. The results showed that low eosinophil count was more likely to occur in patients with ECOG performance status ≥ 2, Currently undergoing anti-cancer treatment, and with accompanying comorbidities. The low eosinophil count group exhibited higher levels of WBC, ANC, PLR, NLR, LDH, hs-CRP, β2-MG, and D-dimer, while exhibiting lower levels of ALC and ALB compared to the high eosinophil count group (Table 2).

**TABLE 2:** Comparison between the low and high eosinophil count groups in patients with lung cancer with omicron.

Characteristics	Low eosinophil count group (*n* = 14)	High eosinophil count group (*n* = 69)	Statistics	*p*-value
Age [years, median (quartile)]	66.9 ± 11.4	61.9 ± 9.6	*t* = −1.726	0.088
Sex, n (%)			χ^2^ = 0.200	0.654
Female	7 (50.0%)	30 (43.5%)		
Male	7 (50.0%)	39 (56.5%)		
Smoking status, n (%)			χ^2^ = 0.200	0.654
Former/current	7 (50.0%)	30 (43.5%)		
Never	7 (50.0%)	39 (56.5%)		
ECOG performance status, n (%)			χ^2^ = 6.761	0.009
0∼1	5 (35.7%)	52 (75.4%)		
≥ 2	9 (64.3%)	17 (24.6%)		
Histological types, n (%)			χ^2^ = 0.516	0.473
NSCLC	14 (100.0%)	62 (89.9%)		
SCLC	0 (0.0%)	7 (10.1%)		
Cancer stage at enrollment, n (%)				0.153*
0- II	2 (14.3%)	26 (37.7%)		
III-IV	12 (85.7%)	39 (56.5%)		
Unknown	0 (0.0%)	4 (5.8%)		
Currently undergoing anti-cancer treatment, n (%)			χ^2^ = 9.084	0.003
Yes	10 (71.4%)	20 (29.0%)		
No	4 (28.6%)	49 (71.0%)		
Number of comorbidities, n (%)			χ^2^ = 9.496	0.002
0	2 (14.3%)	41 (59.4%)		
≥ 1	12 (85.7%)	28 (40.6%)		
Pneumonia/pneumonitis, n (%)			χ^2^ = 0.067	0.795
Yes	8 (57.1%)	42 (60.9%)		
No	6 (42.9%)	27 (39.1%)		
Venous thromboembolism, n (%)			χ^2^ = 0.305	0.581
Yes	2 (14.3%)	4 (5.8%)		
No	12 (85.7%)	65 (94.2%)		
WBC [ × 10^9^/L, median (quartile)]	10.5 (7.2∼13.0)	6.7 (5.0∼8.9)	Z = −2.724	0.006
ALC [ × 10^9^/L, median (quartile)]	0.7 (0.4∼1.1)	1.2 (0.9∼1.7)	Z = −3.067	0.002
ANC [ × 10^9^/L, median (quartile)]	9.1 (6.0∼13.0)	4.4 (3.3∼6.6)	Z = −3.539	< 0.001
PLR [median (quartile)]	256 (162∼380)	189 (135∼257)	Z = −2.007	0.045
NLR [median (quartile)]	14 (6∼25)	4 (2∼6)	Z = −3.296	0.001
ALB [g/L, median (quartile)]	34.7 ± 4.4	38.6 ± 5.9	*t* = 2.335	0.022
LDH [U/L, median (quartile)]	320.0 (248.0∼559.3)	198.0 (182.0∼231.0)	Z = −3.344	0.001
hs-CRP [mg/L, median (quartile)]	24.89 (9.44∼46.26)	3.91 (0.87∼26.06)	Z = −2.688	0.007
β2-MG [μg/mL, median (quartile)]	4.20 (2.06∼5.19)	2.13 (1.65∼2.79)	Z = −2.967	0.003
D-dimer [μg/mL, median (quartile)]	1.27 (0.72∼1.94)	0.59 (0.31∼1.46)	Z = −2.299	0.021

*Fisher’s exact test.

### Disease control and survival rate analysis

Based on the analysis of the impact of eosinophils on the clinical characteristics of patients with lung cancer with omicron, the effect of eosinophils on disease control and survival was further analyzed. The median follow-up time was 251 days (IQR, 229–260) for the high eosinophil count group and 121 days (IQR, 24–257) for the low eosinophil count group.

**FIGURE 4:**
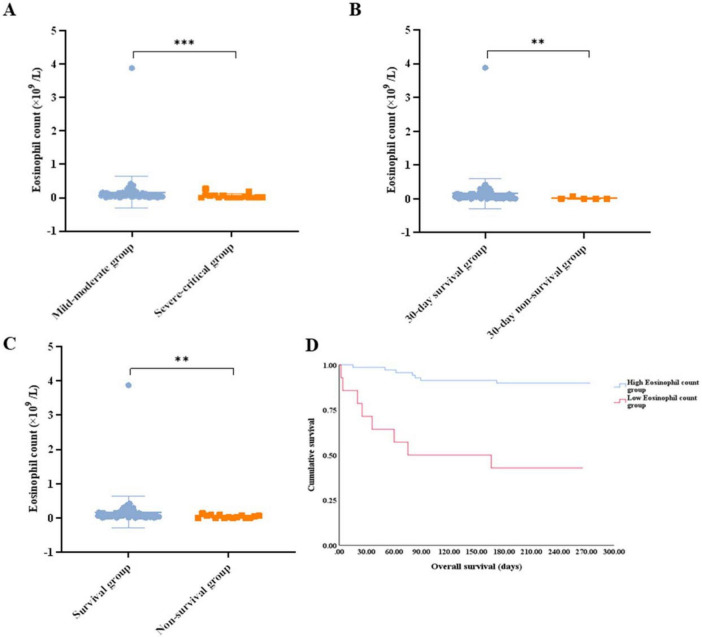
**(A)** Comparison of the eosinophil count between the disease control group and the progressive disease group. **(B)** Comparison of the eosinophil count between patients that survived for 30 days and those that did not. **(C)** Comparison of the eosinophil count between patients that survived and those that did not. **(D)** Survival analysis of the patients with lung cancer with omicron in the high eosinophil count group vs. low eosinophil count group. ** *p* < 0.01; ns, not significant.

Eosinophils between the disease control and the progressive disease groups showed no meaningful difference (0.055 vs. 0.070 × 10^9^/L, *p* = 0.1319) (Figure 4A). To investigate the impact of eosinophils on short-term survival, we examined the relationship between eosinophils and 30-day survival. Our findings revealed that patients who died within 30 days had lower eosinophil counts compared to those who survived during the same period (0.000 vs. 0.070 × 10^9^/L, *p* = 0.0029) (Figure 4B). The eosinophil counts observed during follow-up were significantly lower among patients who died compared to those who survived (0.010 vs. 0.080 × 10^9^/L, *p* = 0.0013) (Figure 4C).

Patients with a low eosinophil count displayed a lower 30-day survival rate (71.4% vs. 98.6%, *p* = 0.002), worse survival (42.9% vs. 89.9%, *p* < 0.001), and shorter median OS (75 days vs. not reached, *p* < 0.001) (Figure 4D). Patients with eosinopenia tended to have lower DCR, although no statistical difference was reached (57.1% for low eosinophil counts vs. 82.6% for high eosinophil counts, *p* = 0.064).

### Eosinopenia was associated with poor outcomes

Compared to the non-omicron group, the omicron group had a higher mortality rate (18.1% vs. 7.5%, *p* = 0.008), especially in the severe-to-critical group (58.8% for the severe-to-critical group vs. 7.6% for the mild-to-moderate group, *p* < 0.001). Therefore, we further investigated the risk factors associated with disease severity and mortality. ROC analysis identified cut-off values of 220.63 and 4.86 for PLR and NLR, respectively.

Univariate logistic regression analysis showed that patients with ECOG performance status ≥ 2, Cancer stage III-IV at enrollment, currently undergoing anti-cancer treatment, with accompanying comorbidities, increased WBC, ANC, NLR, LDH, hs-CRP, β2-MG, and D-dimer levels, and decreased eosinophil count and ALB levels were more serious (Table 3). Further multivariate analysis revealed that ECOG performance status (OR = 6.341, 95% CI: 1.289–31.189; *p* = 0.023), ANC (OR = 13.559, 95% CI: 2.709–67.879; *p* = 0.002), and eosinophil count (OR = 7.365, 95% CI: 1.531–35.433; *p* = 0.013) determinants of the severity (Table 4).

**TABLE 3:** Univariate logistic regression analysis for the severity of patients with lung cancer with omicron.

Characteristics	Variables	OR (95% CI)	*p*-value
Demographics	Age (> 65 vs. ≤ 65 years)	3.007 (0.989 −9.143)	0.052
	Sex (male vs. female)	0.882 (0.303 −2.569)	0.818
	Smoking status (former/current vs. never)	1.134 (0.389–3.304)	0.818
Oncological characteristics	ECOG performance status (≥ 2 vs. 0∼1)	6.233 (1.975–19.672)	0.002
	Histological types of lung cancer (SCLC vs. NSCLC)	0.625 (0.070–5.571)	0.674
	Cancer stage at enrollment (III-IV vs. 0-II)	5.417 (1.139–25.758)	0.034
	Currently undergoing anti-cancer treatment (yes vs. no)	3.286 (1.094 −9.864)	0.034
Comorbidities Complications	Number of comorbidities (≥ 1 vs. 0)	7.179 (1.878–27.450)	0.004
	Pneumonia/pneumonitis (yes vs. no)	2.547 (0.751–8.641)	0.134
	Venous thromboembolism (yes vs. no)	0.763 (0.083 −6.996)	0.811
Laboratory parameters	WBC (> vs. ≤ 10.0 × 10^9^/L)	10.357 (3.069–34.951)	< 0.001
	ALC (< vs. ≥ 1.0 × 10^9^/L)	2.199 (0.738–6.548)	0.157
	ANC (> vs. ≤ 7.0 × 10^9^/L)	16.250 (4.454–59.281)	< 0.001
	Eosinophil count (< vs. ≥ 0.015 × 10^9^/L)	22.143 (5.470–89.641)	< 0.001
	PLR (> 220.63 vs. ≤ 220.63)	2.857 (0.958–8.524)	0.060
	NLR (> 4.86 vs. ≤ 4.86)	7.475 (2.169–25.767)	0.001
	ALB (< vs. ≥ 40g/L)	13.333 (1.670–106.467)	0.015
	LDH (> vs. ≤ 245 U/L)	6.810 (2.142–21.648)	0.001
	hs-CRP (> vs. ≤ 5.0 mg/L)	5.954 (1.562–22.701)	0.009
	β2-MG (> vs. ≤ 2.8 μg/mL)	4.857 (1.578–14.953)	0.006
	D-dimer (> vs. ≤ 1.0 μg/mL)	5.520 (1.717–17.748)	0.004

OR, odds ratio; CI, Confidence interval.

**TABLE 4:** Multivariate logistic regression model for the severity of patients with lung cancer with omicron.

Variables	OR (95% CI)	*p*-value
ECOG performance status (≥ 2 vs. 0∼1)	6.341 (1.289–31.189)	0.023
ANC (> vs. ≤ 7.0 × 10^9^/L)	13.559 (2.709–67.879)	0.002
Eosinophil count (< vs. ≥ 0.015 × 10^9^/L)	7.365(1.531–35.433)	0.013

**FIGURE 5:**
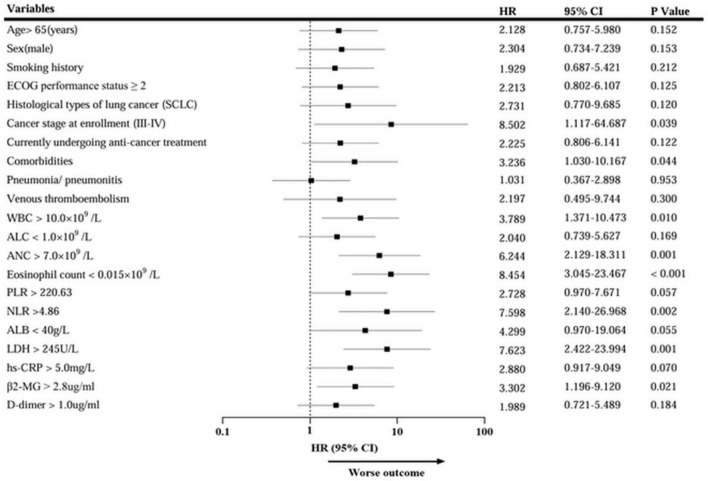
Univariate Cox regression analysis of mortality in patients with lung cancer with omicron. HR, hazard ratio; CI, Confidence interval.

Univariate survival analysis indicated that Cancer stage III-IV at enrollment, comorbidities, increased WBC, ANC, NLR, LDH, and β2-MG, and decreased eosinophil count were associated with an increased risk of death (Figure 5). Further multivariate analysis revealed that histological types of lung cancer (HR = 7.283, 95% CI: 1.626–32.618; *p* = 0.009), ANC (HR = 4.119, 95% CI: 1.289–13.165; *p* = 0.017), and eosinophil count (HR = 7.660, 95% CI: 2.157–27.195; *p* = 0.002) independently correlated with the survival (Table 5). Thus, the eosinophil count was an independent prognostic factor for the severity and survival of patients with omicron, and eosinopenia was obviously associated with poor outcomes.

**TABLE 5:** Multivariate Cox regression model for the mortality of patients with lung cancer with omicron.

Variables	HR (95% CI)	*p*-value
Histological types of lung cancer (SCLC vs. NSCLC)	7.283 (1.626–32.618)	0.009
ANC (> vs. ≤ 7.0 × 10^9^/L)	4.119 (1.289–13.165)	0.017
Eosinophil count (< vs. ≥ 0.015 × 10^9^/L)	7.660 (2.157–27.195)	0.002

## Discussion

This study revealed that patients with lung cancer with omicron had a higher mortality and a lower eosinophil count. In patients with lung cancer with omicron, the cut-off value of eosinophil count for predicting disease severity and death was 0.015 × 10^9^/L, consistent with another study by our team (31). The AUC values were 0.754 for severity and 0.759 for death, respectively, indicating good predictive performance. Further analysis revealed that patients with eosinopenia (< 0.015 × 10^9^/L) had worse 30-day survival and OS. Patients with a low eosinophil count tended to have lower DCR. After multivariate analysis, the eosinophil count was an independent predictor of disease severity and survival, and eosinopenia was significantly associated with poor outcomes.

Since March 2020, we have experienced the COVID-19 pandemic (32). Among patients with lung cancer, the mortality rate of those who developed COVID-19 was as high as 24–39% (1, 12, 33, 34). In this study, the mortality rate of patients with the omicron variant of COVID-19 was 18.1%, which is lower than the mortality rates reported in the aforementioned studies; the difference may be attributed to the variation in COVID-19 variants. Since November 2021, omicron has become the fifth variant of concern, apart from the Alpha, Beta, Gamma, and Delta variants (35). Multiple studies also have revealed that the omicron variant poses a lower risk of mortality compared with other variants (36, 37).

Since the emergence of the omicron variant (B.1.1.529), various sublineages have emerged, such as BA.1, BA.2, BA.4/BA.5 (38), as well as the subsequent omicron XBB sublineage (39), JN.1 sublineage (40), and BF.7 sublineage (41). To date, few studies have explored the role of eosinophils in the infection and disease progression of these sublineages. Zhu et al. (42) analyzed eosinophil counts in 1,157 patients infected with the SARS-CoV-2 omicron/BA.2 variant and found that eosinopenia was an independent risk factor for disease severity (OR 1.34, *p* = 0.006). Another study showed that patients infected with the SARS-CoV-2 omicron/BF.7 variant may also exhibit eosinopenia, although its association with disease severity and clinical outcomes remains unclear (41). Furthermore, some studies analyzing clinical characteristics of SARS-CoV-2-infected patients included those with delta variant but did not specifically examine the relationship between eosinophils and disease severity or outcomes in delta-infected patients (43, 44). Previous research has demonstrated that compared with the parental omicron lineage, each subvariant differs in transmissibility, virulence, and immune evasion ability (38, 40, 45). Omicron variants not only exhibit reduced virulence compared to other SARS-CoV-2 variants but also harbor more mutations and demonstrate stronger immune evasion capabilities (46–48). This study focused on patients with lung cancer who were infected with the omicron variant. Given the dynamic evolution of SARS-CoV-2 variants and the resulting differences in transmissibility, virulence, and immune evasion, the applicability of these findings to other omicron sublineages and earlier variants of SARS-CoV-2 requires cautious interpretation. Continuous monitoring of SARS-CoV-2 variants is warranted to clarify the impact of eosinophils across different strains.

Further analysis in this study revealed that the severe-to-critical omicron increased the mortality of patients with lung cancer (58.8% vs. 7.6%, *p* < 0.001), which was consistent with the results of GRAVID’s study (13). Severe COVID-19 leads to increased severe lung tissue damage (49), a higher proportion of mechanical ventilation, a higher chance of admission to the ICU (13), and a higher likelihood of post-COVID-19 syndrome (14, 50), which eventually leads to irreversible damage to lung function, delayed and reduced tolerance of anti-cancer treatment, and decreased survival.

The prognostic risk factors affecting patients with lung cancer who have COVID-19 have been the subject of numerous studies (1, 12, 13). Several studies have revealed that advanced cancer stages or metastasis at the time of COVID-19 diagnosis (1, 13), accompanying comorbidities (12, 34), and elevated WBC, neutrophil (1, 13), NLR (13, 51), and LDH (13) suggest poor prognosis. Beyond these established factors, our study identified eosinopenia as an independent predictor of disease severity and poor survival in lung cancer patients with omicron infection.

Eosinophils are important innate immune cells that have anti-cancer properties. They can be activated by costimulatory ligands, such as major histocompatibility complex class I (MHC-I), MHC-II, CD80, and CD86. This activation enhances eosinophil-dependent antigen presentation to CD4 + and CD8 + T cells, which promotes T cell proliferation and the release of associated cytokines. Moreover, eosinophils aid in the recruitment of CD8 + T cells by secreting chemokines, such as C-C motif chemokine ligand 5 (CCL5), C-X-C motif chemokine ligand (CXCL) 9, and CXCL10, which enhance the cytotoxic activity of T cells (52). Additionally, eosinophils stimulate the proliferation of natural killer (NK) cells and facilitate the CCL5-dependent recruitment of NK cells to the lungs in mice, contributing to anti-cancer effects (53). Other studies have found that the products of eosinophil lysis may also be involved in tumor destruction (54).

Wang et al. (55) followed 443,542 adults for an average of 5.8 years and found that the eosinophil count in peripheral blood was inversely associated with the occurrence of eight non-hematologic cancers, including lung cancer (aHR range: 0.65–0.95). In addition to being associated with lung cancer development, eosinophils are related to the efficacy of anti-cancer therapies. A study involving 189 patients with NSCLC found that a higher eosinophil proportion after treatment was associated with a better major pathological response (18). In patients with NSCLC treated with immune checkpoint inhibitors, although elevated eosinophils will increase the incidence of checkpoint inhibitor pneumonitis, high eosinophil levels can improve objective response rate and progression-free survival (PFS) (56). An increase in the eosinophil ratio > 1.43 was associated with an improved 5-year PFS and OS (10% vs. 8% and 21% vs. 10%, respectively) in patients with advanced NSCLC who received radiotherapy (57). Moreover, in patients with pleural effusion due to lung cancer, eosinophilia in pleural effusion contributes to improved survival (58). However, the present study revealed that patients with lung cancer with omicron who received current anti-cancer treatment were more prone to develop eosinopenia, which may be connected to the type of treatment and the effect of omicron.

Additionally, eosinophils exhibit antiviral effects. Eosinophils express Toll-like receptors (TLR), including TLR7, to recognize single-stranded RNA viruses (59). Meanwhile, eosinophils can release eosinophil cationic protein (ECP) and eosinophil-derived neurotoxin (EDN) to degrade the viral RNA genome. Eosinophils can also produce cytokines, such as interleukin-12 and interferon-γ, which can express MHC-I and MHC-II, promoting antigen presentation and recruiting virus-specific CD8 + T cells to the lungs to play an antiviral role (60). Besides, Li et al. (61) unexpectedly indicated that Patients with asthma are less susceptible to COVID-19 than the general population. Patients with COVID-19 with asthma had a lower mortality rate than those without asthma (62). Camiolo et al. (63) found a negative correlation between peripheral blood eosinophil count and the expression of angiotensin-converting enzyme 2 (ACE2) in the bronchial epithelium of patients with asthma. ACE2 has a high affinity to the C-terminal domain of SARS-CoV-2 and can promote the entry of SARS-CoV-2 into the cells (64). Therefore, eosinophils may also contribute to the prevention of SARS-CoV-2 infection by downregulating the expression of ACE2.

Numerous studies have shown that patients with severe COVID-19 are more prone to develop eosinopenia (65, 66). Eosinopenia was associated with aggravated chest CT findings (67), the elevated proportion of receiving drug therapy and oxygen support therapy (68), prolonged hospital stay (69), increased risk of admission to ICU (70), increased mortality (68, 71) and the occurrence of Long-COVID (72). Furthermore, the vaccination status of COVID-19 may also affect the level of eosinophils in patients infected with SARS-CoV-2. Li et al. (73) found that compared with vaccinated COVID-19 patients, eosinophil levels in the peripheral blood of unvaccinated COVID-19 patients were lower. Among patients infected with omicron, the third booster of the COVID-19 vaccine showed a more effective and sustained promoting effect on eosinophils, and this effect persisted 5 months after the last vaccination (42). Among patients infected with BF.7, vaccination with two and three doses of the COVID-19 vaccine can reduce the abnormal rate of eosinophils (41). In this study, the influence of vaccination status could not be evaluated due to incomplete vaccination information in the study population. Prospective studies are needed to incorporate patients’ vaccination status and clarify how it influences the role of eosinophils in COVID-19 outcomes.

At present, the reason for the increased susceptibility of patients with severe COVID-19 to eosinopenia is still not apparent. The lung histology of patients who died from severe COVID-19 and developed severe eosinopenia (74) and cytokine profile analysis related to severe COVID-19 (75) have found that depletion and suppression of eosinophil production may play a crucial role. In summary, SARS-CoV-2, especially severe-to-critical cases of SARS-CoV-2, may induce depletion of eosinophils and suppression of their production, leading to eosinopenia in peripheral blood. This will further weaken the antiviral and anti-cancer ability of patients with lung cancer, ultimately leading to cancer progression and reduced survival, which is consistent with our findings. As previously reported in the literature, eosinophilia is positively correlated with the prognosis of lung cancer patients receiving immunotherapy (56, 76). This appears to contradict the above conclusions and the research findings of Lindsley et al. (60). However, this may be related to the fact that immune checkpoint inhibitors (such as PD-1 inhibitors) can relieve immunosuppression, leading to increased secretion of IL-5 by CD4 + T cells. This in turn promotes the recruitment and activation of eosinophils, as well as enhancing eosinophil-mediated cytotoxicity and anti-tumor immunity (77). Another study has shown that increased secretion of granulocyte-macrophage colony-stimulating factor by type II innate lymphoid cells may also be involved (52). The divergent roles of eosinophils in the immunotherapy of COVID-19 and lung cancer reflect the functional plasticity of these cells in different immune microenvironments. However, randomization and large sample clinical studies as well as mechanistic studies *in vivo* and *in vitro* are still needed to explore the impacts of eosinopenia on patients with lung cancer with omicron.

Our study has the following limitations. First, it is a single-institution study with a small sample size, which is not ideal for meeting the per-variable event requirements of multivariate regression. Therefore, we need multi-center, large-sample studies in the future. Second, we have not conducted detailed studies to elucidate the fundamental role and mechanism of eosinopenia in patients with lung cancer with omicron, and we will carry out basic studies to further explore this area. Third, while eosinophils can help predict a patient’s severity and risk of death, incorporating additional features and indicators can enhance the robustness and accuracy of predictive models. Finally, because influenza virus and mycoplasma infections began to occur in China after our follow-up period, we had a short follow-up period for enrolled patients. After that, we will continue to follow the patients for a longer period and further analyze the difference in the impact of omicron and other pathogens on the outcomes of patients with lung cancer.

## Conclusion

This study is the first to systematically analyze the effect of eosinophils on the severity and outcomes of patients with lung cancer with omicron. We found that omicron, especially the severe-to-critical omicron, reduced the survival of patients with lung cancer. Patients with omicron had a lower eosinophil count. Those with eosinopenia tended to have lower DCR, reduced 30-day survival, and decreased OS. The eosinophil count was an independent predictor for disease severity and survival in patients with lung cancer infected with omicron. Eosinopenia was significantly associated with poor outcomes. These insights not only elucidate the pathophysiological role of eosinophils in lung cancer and omicron but also provide valuable guidance for the prevention and surveillance of omicron during the epidemic. Multi-center and large sample clinical studies and *in vitro* and *in vivo* pathogenesis studies are necessary to reveal the mechanism of eosinophils in lung cancer and omicron.

## Data Availability

The original contributions presented in the study are included in the article/Supplementary material, further inquiries can be directed to the corresponding authors.
