# Supplementary material for: Eosinopenia predicts poor outcomes in patients with lung cancer with the omicron variant of COVID-19
Source: Front Med (Lausanne). 2025 Jul 9;12:1583843. doi: 10.3389/fmed.2025.1583843 (PMC12283595; doi:10.3389/fmed.2025.1583843)
Supplement: Supplementary file 1 [file Table_1.docx]

**Table S1** Baseline clinical manifestations, comorbidities, and complications of enrolled lung cancer patients

| **Characteristics** | **Omicron group (n=83)** | **Non-omicron group (n=201)** | **All patients (n=284)** | **p value** |
| --- | --- | --- | --- | --- |
| Fever and respiratory symptoms, n (%) |  |  |  |  |
| Fever (>37.5°C) | 33/83 (39.8%) | 8/201 (4.0%) | 41/284 (14.4 %) | <0.001 |
| Cough | 57/83 (68.7%) | 89/201 (44.3%) | 146/284 (51.4%) | <0.001 |
| Expectoration | 47/83 (56.6%) | 72/201 (35.8%) | 119/284 (41.9%) | 0.001 |
| Dyspnea | 6/83 (7.2 %) | 8/201 (4.0%) | 14/284 (4.9%) | 0.396 |
| Wheezing | 23/83 (27.7%) | 17/201 (8.5%) | 40/284 (14.0%) | <0.001 |
| Chest pain | 13/83 (15.7%) | 13/201 (6.5%) | 26/284 (9.2 %) | 0..015 |
| Comorbidities, n (%) |  |  |  | 0.654 |
| Yes | 40 (48.2%) | 91 (45.3%) | 131 (46.1%) |  |
| No | 43 (51.8%) | 110 (54.7%) | 153 (53.9%) |  |
| Type of Comorbidities, n (%) |  |  |  |  |
| Hypertension | 17/83 (20.5%) | 48/201 (23.9%) | 65/284 (22.9%) | 0.535 |
| Diabetes | 15/83 (18.1%) | 28/201 (13.9%) | 43/284 (15.1 %) | 0.376 |
| Ischaemic heart disease | 12/83 (14.5%) | 27/201 (13.4%) | 39/284 (13.7%) | 0.819 |
| COPD | 6/83 (7.2%) | 11/201 (5.5%) | 17/284 (6.0%) | 0.770 |
| Complications, n (%) |  |  |  |  |
| Pneumonia/ pneumonitis | 50/83 (60.2%) | 45/201 (22.4%) | 95/284 (33.5%) | <0.001 |
| Venous thromboembolism | 6/83 (7.2%) | 9/201 (4.5%) | 15/285 (5.3%) | 0.515 |

COVID-19, coronavirus disease 2019; COPD, chronic obstructive pulmonary disease.
